# Androgen Receptor and Non-Coding RNAs’ Interaction in Renal Cell Carcinoma

**DOI:** 10.3390/ncrna10060056

**Published:** 2024-11-12

**Authors:** Manal A. Hussain, Noha M. Elemam, Iman M. Talaat

**Affiliations:** 1 Research Institute for Medical and Health Sciences, University of Sharjah, Sharjah 27272, United Arab Emirates; u22103877@sharjah.ac.ae; 2 Pure Lab North, Purelab, Abu Dhabi 134808, United Arab Emirates; 3 Clinical Sciences Department, College of Medicine, University of Sharjah, Sharjah 27272, United Arab Emirates; 4 Pathology Department, Faculty of Medicine, Alexandria University, Alexandria 21131, Egypt

**Keywords:** renal cell carcinoma, androgen receptor, non-coding RNAs, urogenital cancers

## Abstract

Renal cell carcinoma (RCC), the most prevalent among the urogenital cancers, accounts for around 3% of new cancer cases worldwide. Significantly, the incidence of RCC has doubled in developed world countries, ranking it as the sixth most common cancer in males, who represent two-thirds of RCC cases. Males with RCC exhibit a higher mortality rate and tend to develop a more aggressive form of the disease than females. Sex-related risk factors, including lifestyle and biological variations, explain this difference. The androgen receptor (AR) oncogenic signaling pathway has been extensively studied among the biological factors that affect RCC. Recent advancements in high-throughput RNA sequencing techniques have underscored the significant roles played by noncoding-RNAs (ncRNAs), previously dismissed as “junk”. The oncogenic potential of AR is manifested through its dysregulation of the ncRNAs’ availability and function, promoting RCC tumorigenesis. This review offers a summary of the most recent findings on the role and molecular mechanisms of the AR in dysregulating the ncRNAs that play a role in the progression of RCC and the possibility of utilizing ncRNAs to target AR as a potential therapeutic strategy.

## 1. Introduction

Renal cell carcinoma (RCC) is the dominant type (>90%) of kidney cancer in adults [1]. It is considered one of the top 10 leading cancers, accounting for 5% of new cancer cases and with 81,610 new cases and 14,390 deaths estimated in the United States in 2024, making it the sixth-highest estimated new cancer cases in males and ninth for females [2]. RCC incidences vary geographically, and they are primarily found in North America and Czechia [3].

RCC is often silent, and its etiology can be attributed to diverse causative factors [4]. In most instances, the incipient stages of cancer are asymptomatic and fortuitously detected during a CT scan, MRI, or ultrasound. The average age at the time of diagnosis is approximately 60 years [5]. Common symptoms are palpable masses, pain, and hematuria, which can manifest in 10% of cases. Typically, the symptoms are ambiguous and appear late. RCC can be inherited or developed because of sporadic genetic changes throughout life, primarily affecting chromosome 3 [6]. Other modifiable factors such as obesity, high blood pressure, alcohol consumption, smoking, history of chronic kidney disease, kidney transplantation, and dialysis increase the risk of developing RCC [7,8].

The renal system consists of two main parts: (1) the renal cortex, where RCC arises, and (2) the renal medulla. The renal cortex is the outer layer of nephrons, the kidney functional units, consisting of the glomeruli in Bowman’s capsule and part of the renal tubule. The remaining tubule extends to the inner renal medulla. RCC begins in the cortex, specifically in the tubular lining epithelial cells, which form around 85% of primary renal neoplasms [9]. One-third of RCC patients develop metastasis, most commonly to the lung (45.2%), bone (29.5%), lymph nodes (21.8%), liver (20.3%), adrenal glands (8.9%), and brain (8.1%) [10,11]. RCC has an overall 5-year survival rate of 85%, which drops to less than 10% in metastatic RCC with poor prognosis [12]. Distinct histological morphologies of cancer cells delineate the most historically prevalent classification of RCC [13].

## 2. Renal Cell Carcinoma Classification

The classical classification of RCC has broadly been categorized into clear and non-clear cell subtypes.

Clear cell renal cell carcinoma (ccRCC) is histologically dominated by clear renal cells that are rich in lipids. It is the most common subtype of RCC overall (70–85%) and the one that is mostly associated with cancer-related death [13]. Tumor cells in ccRCC exhibit von Hippel–Lindau (VHL) tumor suppressor gene loss either by mutation or methylation. VHL is a subunit of the E3 ubiquitin ligase and the key regulator of the hypoxia-inducible factor-1α and -2α (HIF-1α and HIF-2α). HIF-1α and HIF-2α are oxygen-sensitive transcription factors which are ubiquitylated and degraded by VHL at normal oxygen levels. On the contrary, HIF-1α and HIF-2α stabilize in the event of increased HIF gene transcription, when VHL function is lost, or under hypoxic environments. This, in turn, leads to the upregulation of HIF target gene expression, including vascular endothelial growth factor (VEGF), platelet-derived growth factor (PDGF), and transforming growth factor alpha (TGF-α). The increased expression of growth factors promotes vascularization, thereby improving cellular access to oxygen and nutrients that are essential for survival. This phenomenon is integral to embryonic development and tumorigenesis-associated characteristic “angiogenesis” [12,13].

The histological subtypes characterized by features of non-clear cell RCC (nccRCC) constitute a proportion ranging from 20% to 25% within the spectrum of RCC cases [12]. The most common nccRCC subtype is papillary renal cell carcinoma (pRCC), which forms 15–20% of incidences. It is a heterogeneous disease with a better prognosis than ccRCC [14]. Classically, pRCC was subdivided into types I and II. Type I is generally multifocal, characterized by cribriform papillae enveloped with a single small layer of cells with uniform nuclei and small oval nucleoli. On the other hand, type II appears in solitary lesions and contains less organized papillae covered by pseudostratified large cells with large round nuclei and prominent nucleoli. Type I has a slower growth rate and better prognosis, whereas type II tends to metastasize earlier [15,16]. The gene mutation of mesenchymal-epithelial transition factor (MET), a tyrosine kinase receptor, was found in hereditary pRCC and 13% of sporadic forms of pRCC, in addition to MET mutation being more prevalent in type I compared to type II. Further investigations of pRCC have identified significant chromosomal rearrangements and numerical instabilities due to several gene mutations involved in chromatin modification and cell cycle regulation. The conventional classification system seems to exhibit limitations, and current studies suggest integrating the cancer genotype with its phenotype, thereby affording a more accurate and refined classification [14].

Chromophobe renal cell carcinoma (chRCC), constituting approximately 5–7% of all RCCs, ranks as the third most common RCC and is the second most prevalent among nccRCCs. ChRCC is primarily sporadic but can be associated with multiple genetic disorders like Birt–Hogg–Dubé syndrome (BHD) [17,18]. BHD is linked to germline mutation in the tumor suppressor gene folliculin (FLCN); however, FLCN alteration is an inconsistent characteristic of chRCC [18]. The most common tumor in BHD patients is the hybrid oncocytic/chromophobe tumor (HOCT). The other rare autosomal dominant disorders associated with chRCC include BAP1 tumor predisposition syndrome (BAP1 gene) [19], hereditary paraganglioma/pheochromocytoma (PGL/PCC) syndrome (SDH A/B/C/D genes) [20], Cowden syndrome (PTEN mutations) [21], and tuberous sclerosis (TSC1/2 mutations) [22]. The loss of chromosomes 1, 2, 6, 10, 13, 17, and 21 indicates genetic signatures of classic and eosinophilic chRCC. On the other hand, chromosomal gains are linked to sarcomatoid chRCCs [23,24]. This cancer is distinguished by a highly favorable prognosis within the spectrum of RCCs. It boasts a higher overall survival rate, has a diminished probability of developing metastatic characteristics, and exhibits a less aggressive pathological morphology. Based on morphological differences, chRCC is categorized into the classic and eosinophilic subtypes. However, diagnosing the eosinophilic subtype is challenging due to its features resembling those of oncocytoma, a benign tumor [23]. Microscopically, chRCC presents sizable pale cells, a network-like cytoplasm, and a cell nucleus surrounded by a halo [25].

Multilocular cystic renal cell carcinoma or multilocular clear cell renal cell carcinoma (mcRCC) is a rare cancer comprising 1–4% of cases, that is predominant in males (men/women ratio 3:1), and which can be curable and has a better overall survival rate. It appears first as cysts with a gelatinous or hemorrhagic nature, covered by a single layer of epithelial cells with clear cytoplasm and a small nucleus. The tumor is usually confined in the renal capsule [13,26]. An additional prevailing subtype observed in males is collecting duct renal cell carcinoma (cdRCC), which constitutes a rarity, accounting for less than 1% of all cases within this subtype. CdRCC is characterized by its heightened aggressiveness, with approximately one-third of cases being diagnosed at the metastatic stage, resulting in a notably inferior prognosis compared to ccRCC. Originating within the collecting duct, cdRCC manifests distinct histological attributes, including a tubular pattern, nuclear enlargement, and other features that are indicative of high-grade malignancy. The definitive diagnosis of cdRCC necessitates a meticulous exclusion of other RCC categories through the use of molecular markers [13,27].

RCC exhibits a gender bias, with male cases reported twice as frequently as female cases [7]. Male patients often experience lower overall survival, worse prognosis, and worse tumor progression. These findings point to the potential role of gender-related factors, whether modifiable environmental factors or biological variations, in highlighting the importance of RCC risk. Additionally, 15–20% of patients diagnosed with kidney cancer are smokers [28]. Despite a decrease in tobacco use over the previous few decades, males were still found to continue smoking more frequently than females [29]. Moreover, males are exposed to carcinogens more frequently than females because of the differences in their work nature. On the other hand, non-modifiable factors, such as sex hormones, seem to play a vital role in cancer. For instance, androgens, which are present at higher levels in males, are associated with the promotion of cancer, including RCC. Conversely, females exhibit higher levels of estrogen hormones, which appear to provide a protective effect against cancer [30].

## 3. Non-Coding RNAs (ncRNAs)

Non-coding RNAs (ncRNAs) are RNAs that do not code for proteins and account for 98% of the human transcriptome output [31]. In addition to the housekeeping ncRNAs (tRNA, rRNA, and mRNA), there is another group called regulatory ncRNAs, which have long been assumed to be junk. However, RNA-sequencing and other developed bioinformatics tools have shown that ncRNAs play a crucial regulatory role, including suppressing or enhancing the tumor suppressor genes and oncogenes, respectively [32].

### 3.1. MicroRNAs (miRNAs)

MiRNAs are the most extensively studied ncRNAs, with short lengths ranging from 17 to 25 nucleotide bases. MiRNA biogenesis is categorized into canonical and non-canonical pathways. In canonical pathways, the biogenesis starts in the nucleus when Drosha cleaves the pri-miRNA hairpin, generating precursor miRNA (pre-miRNA). The pre-miRNA is then transported to the cytoplasm, where the Dicer enzyme processes it to produce mature miRNA. On the other hand, non-canonical biogenesis begins with 7-methylguanine-capped (m7G) pre-miRNA or small hairpin RNA (shRNA) that is cleaved in the nucleus and exported to the cytoplasm for further processing by Dicer-dependent or -independent maturation. In all pathways, mature miRNAs are loaded into the Argonaute proteins, which form miRNA-induced silencing complex (miRISC) [33]. MiRNAs function at the post-transcription level via binding to the 3′ untranslated region (3′ UTR) of the mRNA-making complement strands, where the degree of complementarity determines the miRNA-blocking mechanism of the target gene, either by translation repression or mRNA degradation. At least half of the protein-coding genes are regulated by miRNAs, making them pivotal in cancer development due to inducing cell proliferation, invasion, and metastasis. Some miRNAs that act as tumor suppressors were found to be reduced in cancer, while the opposite was reported for oncogenic miRNAs, making them promising targets for cancer and potential diagnostic biomarkers due to their availability in the body’s fluids [34].

### 3.2. Circular RNAs (CircRNAs)

Circular RNAs (circRNAs) are another type of endogenous ncRNA with a distinctive structure of covalently closed single strands, making them more durable and resistant to RNAases [35,36]. Initially, scientists thought they were a plant pathogen called a viroid [37]. Later, it was discovered that they are a common cell feature in many species, including humans. CircRNAs are mainly composed of exon stretches of protein-coding genes, but they can also be intronic, intergenic (at the 3′UTR or 5′UTR), or antisense RNAs [38,39]. CircRNAs are synthesized by spliceosomes through pre-mRNA back-splicing [40] and have three biosynthesis models: (1) exon skipping, (2) intron pairing, and (3) RNA-binding protein-driven circularization [41]. They mainly function as a “sponge” for miRNAs and proteins [42,43]. For miRNA regulation, circRNAs contain a complementary sequence to that of the forming miRNA, and they bind to form a “reservoir” that adjusts miRNA availability and stability [44,45]. CircRNA–protein interaction seems more intricate, regulating protein expression, formation, availability in the subcellular location, and pathological mechanisms. CircRNA–protein interaction is pivotal in governing circRNA production and breakdown [42,43].

### 3.3. Long ncRNAs (LncRNAs)

Long ncRNAs (lncRNAs) are long RNA sequences of around 200 nucleotides that were initially thought not to code for protein. They have a 5′ cap and a 3′ poly-A end like mRNA, and are expressed in all organisms [46]. They are differentially transcribed in the tissues and from different genomic regions such as enhancers, promotors, intergenic regions, and primary transcripts [47,48]. The biosynthesis of lncRNAs happens either by canonical splicing or a non-canonical mechanism that is controlled by epigenetic modifications like acetylation and methylation [46]. LncRNAs are subdivided based on different factors, like their location in protein-coding genes (PCGs): (1) intergenic, where the lncRNA sequence is located between two PCGs [49]; (2) intragenic in the intron region of the gene; (3) sense in the forward strand extending across many introns and exons; (4) antisense in the reverse strand; and (5), for bidirectional lncRNAs, transcribed in the opposite direction to the promoter on the sense strand [49,50]. Also, another classification could be based on their function (tRNA, cRNA, and rRNA) or their cellular location (nuclear, mitochondrial, and cytoplasmic) [49,50]. LncRNAs have various functions that interact with different molecules, including DNA, mRNA, proteins, and miRNAs [50]. They act as signaling molecules, regulating different pathways and inducing epigenetic modifications [51]. Decoy lncRNAs act as transcription factors and miRNA sponges to regulate gene expression, while scaffold lncRNAs allow chromosomal reshuffling and translation initiation. LncRNAs affect multiple pathological and physiological processes by influencing the timing and level of gene expression. LncRNAs were found to be dysregulated in many cancers, such as renal cancer, via altering multiple oncogenic pathways, making them novel biomarkers for cancer diagnosis and promising therapeutic tools [46].

This article provides a comprehensive overview of the interplay between the AR and ncRNAs in the progression of RCC, with them being involved in regulating tumor initiation, proliferation, invasion, and metastasis. It also discusses the potential of targeting the AR through ncRNAs as a potential treatment approach.

## 4. Non-Coding RNAs as Biomarkers in RCC

Several ncRNAs have emerged as potential biomarkers for RCC due to their roles in tumor progression, metastasis, and response to treatment. For example, miR-21 was found to be upregulated in RCC, being associated with tumor progression and poor prognosis [52,53]. At the same time, miR-210 was linked to hypoxia-induced pathways and served as a marker for aggressive RCC [54]. The miR-200 family was shown to play a role in epithelial-mesenchymal transition (EMT), with miR-200c often downregulated in RCC [55]. Regarding circRNAs acting as biomarkers, circHIAT1 is known to suppress RCC metastasis. It shows potential as a diagnostic and prognostic marker [56], while circEGLN3 promotes RCC cell proliferation and migration, making it a candidate for disease progression markers [57]. The lncRNA HOTAIR was associated with poor prognosis and advanced stages in RCC [58,59]. MALAT1 was linked to RCC metastasis and was often found to be overexpressed, correlating with poor clinical outcomes [60,61]. Similarly, PVT1 was upregulated in RCC and contributed to tumor growth, making it a potential marker for aggressiveness [58,62]. Therefore, ncRNAs show promise for early detection, prognosis, and treatment response monitoring in RCC, although further validation is necessary for their clinical use. Other ncRNAs include piRNAs and tRNAs, which could also be involved in RCC pathogenesis, potentially influencing processes such as genome stability, translation regulation, and cell signaling [63,64].

## 5. Androgen Receptor (AR) and its Tumorigenic Role in RCC

Androgens are male sex hormones such as testosterone and its metabolite dihydrotestosterone (DHT) that have a crucial role in early embryonic development, expressing and maintaining male physiology. They have been extensively studied in hormonal pathways due to their involvement in the pathological processes of many diseases, including cancers. For instance, hyperandrogenism can cause polycystic ovary syndrome and cancers, while androgen deprivation is one of the standard treatments for advanced malignancies such as prostate cancer, leading to a notable improvement in survival rates [30,65]. The biological function of androgens is primarily mediated by their binding to the androgen receptor (AR) or the steroid hormone receptor. Androgens ligand binding to the AR induces homodimerization and nuclear localization, acting as a transcription factor. Once in the nucleus, the AR regulates gene expression via direct binding to the DNA at androgen response elements (AREs) [66] or through a non-DNA-binding signal [67]. Recently, attention has been drawn to the regulatory effect of the AR via its interaction with non-coding RNAs in the progression of various tumor types, including breast, lung, prostate, and renal cancers [12].

The AR has shown a favorable role in initiating and promoting RCC. Multiple investigations were involved in introducing ARs into both normal kidney epithelial cells and renal cancer cells and assessing the downstream processes. Studies have revealed that normal cells transfected with ARs exhibited a heightened ability to form colonies of larger size and an increased proliferation rate. This was further validated by introducing ARs into RCC cell lines (769P, 786-O, OSRC-2, and ACHN), which elicited enhanced cell growth compared to the control. Further, the proliferation was reversed in those cells upon treatment with anti-androgen [68]. Additionally, suppressing the AR in the SW839 RCC cell line using siRNA reduced cell growth. The AR also increased the RCC cell migration rate, as determined using the transwell migration assay [68]. The *in vivo* findings were consistent, indicating that introducing the renal carcinogen Fe-NTA (ferric nitrilotriacetate) transformed cells into xenograft models and accelerated the tumor growth rate [68,69]. Furthermore, the analysis observed genes related to tumor invasion and metastasis to be upregulated in AR-transfected cells, including Kai-1, SYK, Serpinb, HIF2a, and VEGF, critical players in RCC tumorigenesis [68]. On another note, a positive correlation was reported between heightened AR levels and increased expression of the VHL tumor suppressor, hinting at an alternative mediator through which AR acts [68]. Interestingly, further investigations showed that the AR could act through the modulation of ncRNAs. In RCC, the AR stimulates angiogenesis by inducing endothelial cell proliferation through the AKT → NF-κB signaling pathway [70]. While degrading activated ARs, it restored sensitivity to sunitinib receptor tyrosine kinase inhibitors (RTKIs) [71], an effective treatment for ccRCC patients [72].

## 6. AR Targeting the ncRNAs in RCC

### 6.1. AR Targeting miRNAs

A negative relation between miRNA-145 and the AR was observed, where significantly reduced miRNA-145 levels were associated with increased AR levels on RCC cell lines. It was suggested that miRNA-145 acts as a tumor suppressor by suppressing the expression of HIF-2α and VEGF, as well as upregulating AR-suppressed miRNA-145, by binding to AREs in the miRNA-145 gene at the promoter region. The miRNA promoter region also contains the p53 response element (p53RE), where p53 binds to miRNA and promotes miRNA-145 expression. Therefore, such findings highlighted the AR as a secondary factor in promoting the HIF-2α/VEGF signaling pathway in RCC, leading to enhanced tumor invasion independently of VHL [68,69].

Interestingly, dysregulation of the AR has been implicated in modulating tumor metabolism. Downregulated arginosuccinate synthase 1 (ASS1) was considered a tumorigenesis characteristic, promoting the proliferation of many cancers, including breast cancer and renal cancer [73,74]. ASS1 is an enzyme that synthesizes the non-essential amino acid arginine. Suppressed ASS1 levels in cancer results in tumor auxotrophy (a tumor dependent on extracellular arginine) and is associated with poor outcomes in many cancers [73,75]. Studies using immunohistochemical staining showed that the ASS1 levels in RCC tissues appeared lower than in adjacent normal renal tissues [74]. Further, researchers have suggested that the AR’s role in ASS1 downregulation is to promote RCC. This negative relation has been proven by silencing the AR using AR-shRNA in SW-839 and OSRC-2 cells, which increased ASS1 expression. On the other hand, AR amplification using AR-cDNA in A498 and OSRC-2 cell lines resulted in decreased ASS1 at the protein level and minor changes at the transcriptional level [76]. This inconsistency in protein and transcript levels implies a potential role for ncRNAs, which could act on ASS1 at the post-transcription level, in the treatment of RCC. Research has found that ASS1 has 12 pseudogenes [74]. In cancer, it has been shown that pseudogenes can regulate gene expression through their ability to act as miRNA sponges [35]. Investigations have revealed that ASS1 pseudogene 3 (ASS1P3) is indirectly responsible for ASS1 regulation, and blocking ASS1P3 has influenced ASS1 expression. The same study identified that ASS1P3 acts through miR-34a-5p, as ASS1P3 sponges miR-34a-5p, blocking its ability to silence the target ASS1 [74].

Moreover, the AR was found to bind to ASS1P3 directly, suppressing ASS1P3′s sponging ability. ASS1P3 function loss leaves miR-34a-5p free, which binds to the ASS1 mRNA transcript and blocks its ability to produce protein. Such results were confirmed using an orthotopically xenografted mouse model and OSRC-2 renal cancer cell line with an over-expressed AR (OE-AR), overexpressed ASS1P3 (OE-ASS1P3), or both (OE-AR-ASS1P3). The larger tumor weight was reported in OE-AR. Conversely, OE-ASS1P3 showed a reduced tumor size and weight, aligning with its function as a sponge for miR-34a-5p oncogene, subsequently enhancing the expression and activity of ASS1. On the other hand, in the OE-AR-ASS1P3 model, it seems that the AR blocked ASS1P3’s sponging ability, allowing miR-34a-5p interaction and the inhibition of ASS1, therefore increasing tumor growth. Such data showed that the AR can promote RCC growth via miR-34a-5p alteration [74].

Evidence suggests that AR’s role is not limited to the initiation, proliferation, and invasion of RCC, and that it can also manipulate the metastasis route and destination by ncRNA modulation. Metastasis is the process in which cancer cells spread beyond the primary location, most commonly to the lung and bone in RCC. Approximately 33% of patients diagnosed with RCC develop metastasis, either at the time of diagnosis or following nephrectomy [10]. Based on an epidemiological survey conducted on ccRCC patients at the Chinese PLA General Hospital, a gender ratio of 4.9:1 male to female has developed lung metastasis, indicating the potential involvement of hormones [77]. Metastasis can occur by cancer cell’s intravasation either through the blood circulatory system (hematogenous metastasis, “HM”) or the lymphatic vessels (lymphogenous metastasis, “LM”). RCC primarily spreads through the bloodstream to the lungs. Cancer cells’ decision to disseminate and their routes depends on multiple factors, including the tumor cell’s characteristics, the tumor’s surrounding microenvironment, differences in gene expression, and the expression of various receptors and signaling molecules. For instance, tumor necrosis factor-alpha (TNF-α), transforming growth factor-beta (TGF-β), matrix metalloproteinases (MMPs), interleukins 6, 8, and 10 (IL-6, IL-8, IL-10), and VEGF-A are known to support the HM pathway. In contrast, VEGF-C and VEGF-D are categorized as factors that aid LM [77]. Studies have revealed AR involvement in promoting the RCC metastasis route through the modulation of the angiogenesis-related factors HIF-2α and VEGF-A against the lymphangiogenesis factors VEGF-C and VEGF-D [78]. Treating RCC cell lines with androgen and DHT has increased the HIF-2α-VEGF-A at both the mRNA and protein levels but decreased the VEGF-C at the protein level [77]. On the contrary, silencing the AR had the opposite effect. To further confirm the AR’s role in the fate of the metastasis destination, the RCC A498 cell line, with relatively low AR expression, was used. The induction of the AR in A498 cells enhanced hematogenous endothelial cell tube formation but reduced the lymphatic endothelial cell tube. In contrast, an opposite pattern was reported upon AR silencing in SW839 cells [77].

Further investigations have focused on the potential miRNA candidate through which the AR acts. It was found that AR knockdown has reduced miR-185-5p sharply. In addition, the possible mechanism of miR-185-5p’s regulatory role might be promoting VEGF-A through binding to the 5′ promoter of HIF-2α and blocking VEGF-C by binding to the 3′ UTR region concomitantly. Also, increased AR expression was positively correlated with HIF-2α and VEGF-A, thus enhancing the tumor cells’ invasion through hematogenous spread [77].

### 6.2. AR Targeting CircRNAs

While AR overexpression in RCC has been associated with lung metastasis, the most prevalent metastatic site in RCC, a study indicates that the AR may attenuate RCC bone metastasis (RBM) through manipulation of the expression of circRNAs [79]. The AR expression at the mRNA and protein levels was oppositely correlated with RBM when compared with non-RBM cancer [79]. Further, increased osteolytic formation was linked with bone metastasis. It has been discovered that treating primary bone marrow cells from mice (BMMs) with conditioned media from an AR-upregulated RCC cell line decreased the prevalence of osteolytic formation. In contrast, the opposite effect was observed in BMMs treated with conditioned media collected from AR-downregulated cells. This indicates that the AR inhibits osteolytic formation and decreases the likelihood of RBM in BMMs [79]. Previous studies have shown that circRNAs play a critical role in the conversion of cells to osteoblastic or osteolytic states [80]. CircRNAs are single-stranded RNAs that have a variety of roles in gene regulation, including miRNA’s sponging capacity [80,81]. Initially, they were categorized as ncRNAs, but later studies have revealed their surprising ability to be translated into proteins [82]. CircRNAs were identified to have potential as a signature biomarker for cancer detection and diagnosis, as a therapeutic agent for targeting the oncogene, or as a therapeutic target [83]. A study by Dongkui Gong and his colleagues examined the expression of dysregulated circRNAs to comprehend how the AR inhibited the formation of osteolytic aggregates [79]. CircRNA_0092355, or so-called circEXOC7 (as it is derived from the EXOC7 gene), was reported to be downregulated in non-RBM patients compared to other RCC patients [79]. On the other hand, increasing the circEXOC7 expression led to blocked AR expression and promoted osteolytic formation and proliferation. It was noteworthy that the circEXOC7 transcription was unaffected by enhanced AR expression [79].

Previous investigations have highlighted that circRNA production can be controlled by RNA-binding proteins at the post-transcriptional level [84,85]. The impact of the AR was tested on one of the potential circRNA regulator proteins, DHX9, and the findings showed a positive relationship between the levels of DHX9 and the AR expression [79,86]. One possible explanation could be that the AR binds to AREII in the DHX9 promoter region, which makes the DHX9 expression higher. DHX9 negatively regulated the production of circEXOC7, which suppressed its sponging ability and freed up its target miRNAs, such as miR-149-3p. In this study, miR-149-3p acted as a regulator that helped prevent the deuteriations of bone tissue that often occur in metastatic tumors by diminishing the function of colony-stimulating factor 1 (CSF1), which is recognized for its role in promoting bone degradation. Therefore, AR could inhibit bone metastasis via DHX9/circEXOC7/miR-149-3p/CSF1 axis regulation [79].

Other candidate circRNA levels were found to be altered by AR, which thus promoted RCC migration and invasion. For instance, AR overexpression correlated with downregulation of the hipocampus abundant transcript-1 circular RNA (circHIAT1) [87]. CircHIAT1 RNA, originating from the HIAT1 gene, was first discovered in the mammalian brain and produces a transmembrane protein involved in spermatogenesis [45]. CircHIAT1 demonstrated anti-cancer properties by suppressing cell growth, proliferation, migration, and epithelial-mesenchymal transition (EMT). Its levels are often diminished in various cancers, including gastric cancer, hepatocellular carcinoma, and prostate cancer, and are correlated with poorer survival rates [45,88,89]. The AR altered the circHIAT1 levels in RCC by binding to the AREs located in the 5′ promoter region upstream of the transcription initiation point of the HIAT gene, decreasing its transcription level, which appeared to be linked with the overexpression of CDC42. CDC42 is a Rho family GTPase and its overexpression could promote cell division; therefore, it plays a crucial role in cancer metastasis [90]. The expression of CircHIAT1 was higher in patients with non-metastatic ccRCC and was correlated with better overall survival [87].

### 6.3. AR Targeting LncRNAs

In genitourinary cancers, lncRNAs participate in multiple pathways, such as tumor angiogenesis, proliferation, apoptosis, and the emergence of drug resistance [91]. On the other hand, they can also be targeted and suppressed by carcinogenic pathways to promote cancer development. A study has revealed that the suppression of lncRNAs by the AR can increase vasculogenic mimicry (VM) formation in RCC [92]. VM, a novel form of angiogenesis, involves cancer cells organizing themselves into tube-like structures independently of endothelial cells [93]. VM was identified after extensive research prompted by the unsatisfactory outcomes of targeting classical angiogenesis in cancer treatment [94]. Initially, AR’s positive association with VM formation was supported experimentally using RCC cell lines and the immunohistochemical staining of RCC patients’ samples. Further investigations revealed higher VM levels in RCC patients at stages II and III compared to stage I, with a higher occurrence rate in males than females. To understand the AR–VM molecular mechanism in RCC, VM-related genes were screened, and it was found that the Twist1 VM-related gene was significantly influenced by AR dysregulation. Increased expression of the AR correlated with elevated levels of Twist1 mRNA and protein, as confirmed using qRT-PCR and western blot, respectively [92]. Twist1, a transcription factor that modulates embryogenesis, is considered a prognostic marker in hepatocellular carcinoma cells, as it is associated with stimulating EMT, VM formation, and migration [95]. Similarly, Twist1 was linked to a higher VM occurrence, lower survival rates, later disease stages, and poor prognosis in RCC [92,96]. Moreover, Twist1’s prolonged stability suggested a potential interference from ncRNAs. This led to the discovery of Twist1 associated long non-coding RNA regulated by AR (TANAR), which was found to bind at the 5′ UTR of Twist1 mRNA, in competition with the mRNA decay factor upstream frameshift 1 (UPF1), and inhibit its activity [92]. HOTAIR lncRNA was found to be involved in angiogenesis [97], where it was found to interact with AR, stimulating the transcription activity of GLI2, a hedgehog pathway mediator. GLI2 is commonly dysregulated in ccRCC and negatively linked to the overall survival of patients [97,98]. Furthermore, HOTAIR was found to promote RCC cell proliferation and growth both in vitro and in vivo. In clinical RCC samples, HOTAIR expression was inversely correlated with Salvador homolog 1 (SAV1) expression. Using cell lines, HOTAIR was found to downregulate SAV1 by directly binding to the SAV1 protein and increasing histone H3K27 methylation [59].

## 7. Targeting AR: A Promising Therapeutic Strategy

Androgen deprivation therapy is a promising approach in RCC in which targeting the AR in RCC sensitizes the cells to RTKIs; one possible mechanism for this is through the hedgehog or PI3K signaling pathways [71]. Using a lncRNA candidate, AR protein can be targeted, hence reversing the AR tumorigenesis effect on RCC [99]. The lncRNA termed suppressing androgen receptor in renal carcinoma (SARCC) showed promising results both in vitro and in vivo [100]. Experiments proved that increasing lncRNA-SARCC reduces AR protein and AR target gene expression. This was achieved by lncRNA-SARCC direct binding to the AR protein, preventing its nuclear localization. To link the reduced expression of lncRNA-SARCC in RCC with cancer aggressiveness, lncRNA-SARCC was knocked down in SW839 and OSRC-2, which resulted in a change in cells’ physical appearance, increased invasion, and increased migration capability [100]. As miRNAs’ deregulation is common in RCC, possible miRNAs were screened for further dissection of the molecular mechanism of lncRNA-SARCC–AR interaction [101]. The tumor-suppressing miR-143-3p exhibited a notable response to the lncRNA-SARCC and AR alterations, as it possesses AREs in its promoter region, indicating the involvement of the AR as a transcription factor that binds to suppress its activity. Additionally, increased the AR expression in RCC was reported to have repressed miR-143-3p, causing the loss of its regulatory effect on K-RAS, P-ERK, and AKT, the commonly activated pathways in cancer [99,100]. MiR-143 silences the K-RAS oncogene by binding to the 3′ UTR of its transcript, thereby reducing its protein expression. K-RAS, a small GDP-/GTP-binding protein and intracellular signal transducer, upon recruitment and activation, phosphorylates and activates multiple downstream oncogenic signaling pathways [102], including the RAF, MAPK, and ERK cascade pathways, which are phosphorylated and translocated to the nucleus to stimulate the expression of cell proliferation genes [103]. Similarly, K-RAS can activate the PI3K, Akt, and mTORC1 pathways [104], with mTORC1 subsequently activating the HIF-1α and c-Myc transcription factors [105]. AR protein disruption by lncRNA-SARCC inhibits the proliferation of VHL-mut RCC cells (not VHL-normal) by suppressing the HIF-2α/c-myc oncogenic pathway [106].

Another therapeutic approach to target the AR in RCC tumorigenicity could be the AXL/c-Met pathway. c-Met, a receptor tyrosine kinase (RTK), and its downstream pathway are implicated in RCC progression, and high c-Met expression in bone metastatic lesions was associated with poor prognosis [107]. Previous studies of prostate cancer reported an inverse correlation between c-Met expression and AR expression [108]. It was suggested that increased c-Met expression was related to disease progression and resistance to androgen deprivation therapy [109]. In addition, recent findings indicate that cabozantinib (Cabo) inhibits c-Met and a few other RTKs, including AXL, and is approved for treating patients with advanced-stage RCC. Also, targeting AXL along with c-Met inhibition was beneficial in preventing acquired therapeutic resistance in RCC [110].

## 8. Conclusions

**Figure 1:**
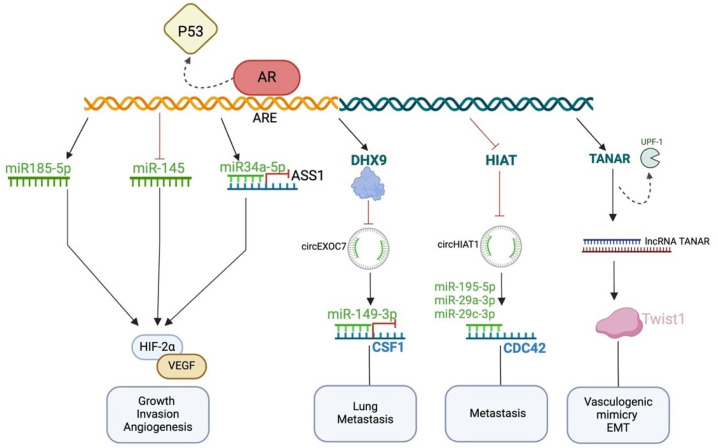
Oncogenic effect of increased AR expression in RCC through the modulation of ncRNAs. AR: androgen receptor, ARE: androgen response element, ASS1: arginosuccinate synthase-1, HIF-1α: hypoxia-inducible factor-1α, VEGF: vascular endothelial growth factor, CSF1: colony-stimulating factor-1, CircHIAT1: hipocampus abundant transcript-1 circular RNA, TANAR: Twist1 associated long non-coding RNA, UPF1: regulated by AR, up-frameshift protein1, EMT: epithelial–mesenchymal transition.

Research has indicated the oncogenic role of the AR in RCC, altering numerous types of ncRNAs and signaling pathways including the miR-145, miR-185-5p/HIF-2α/VEGF, miR-34a-5p/ASS1, DHX9/circEXOC7/miR149-3p/CSF1, circHIAT1/miR-195-5p, miR-29a-3p and miR-29c-3p/CDC42, and TANAR/UPF1/Twist1 pathways [69,74,77,79,87,92] (Figure 1). Inhibiting the excessive expression of the AR holds significant promise in attenuating RCC’s initiation, proliferation, invasion, and metastasis, along with alleviating resistance to sunitinib, a current therapeutic approach to metastatic RCC that uses tyrosine kinase receptor inhibitors. Such an objective can be pursued through various approaches, including the modulation of ncRNAs [100]. The further validation of ncRNA–AR interaction by exploring more patient samples is required. The dysregulated levels of ncRNAs in RCC can serve as promising diagnostic and prognostic biomarkers and can be used for targeting tumor growth and dissemination, and overcoming drug resistance. NcRNAs are present in blood serum and plasma in a stable form, thus facilitating cancer detection through feasible techniques such as liquid biopsy. NcRNAs can also be used as a prognostic biomarker, enabling the anticipation of tumor behavior and aggressiveness. Targeting the AR with ncRNAs, especially lncRNAs, holds great promise and challenges. Cancer is heterogeneous, and the ncRNA abundance and activity vary in a tissue-dependent manner. It is also affected by the previously given treatment, patient age, patient gender, and disease stage. Other challenges in this therapeutic approach include understanding the functions of ncRNAs, efficient delivery to the tumor cells, and the need for more specificity, called ‘off-target’. Despite these obstacles, researchers are pushing forward in this field with rapid technological advancements, offering hope of surpassing these limitations [111].

Further research is needed to clarify the mechanisms of AR regulation by ncRNAs beyond canonical pathways, such as potential interactions with non-genomic AR signaling or crosstalk with other oncogenic pathways. Additionally, optimizing the therapeutic targeting of the AR through ncRNAs raises challenges around delivery specificity, minimizing off-target effects, and understanding patient-specific variables like tumor heterogeneity and prior treatment history. Exploring these areas will be essential to fully harnessing the potential of ncRNAs as diagnostic, prognostic, and therapeutic tools in RCC [112]. Addressing these challenges could pave the way for novel, more effective therapeutic strategies and personalized treatment approaches.
